# Daily Nutritional Dose Supplementation with Antioxidant Nutrients and Phytochemicals Improves DNA and LDL Stability: A Double-Blind, Randomized, and Placebo-Controlled Trial

**DOI:** 10.3390/nu5125218

**Published:** 2013-12-18

**Authors:** You Jin Kim, Yoon Hee Ahn, Yeni Lim, Ji Yeon Kim, Joohee Kim, Oran Kwon

**Affiliations:** 1 Department of Nutritional Science and Food Management, Ewha Womans University, Seoul 120-750, Korea; E-Mails: eugene841226@gmail.com (Y.J.K.); ayh181@naver.com (Y.H.A.); yeni0223@hanmail.net (Y.L.); 2 Department of Food Science and Technology, Seoul National University of Science and Technology, Seoul 139-743, Korea; E-Mail: jiyeonk@seoultech.ac.kr; 3 BioFood Network, Ewha Womans University, Seoul 120-160, Korea; E-Mail: wynona@biofood.or.kr

**Keywords:** multi-micronutrient supplement, nutritional dose, phytochemicals, human intervention

## Abstract

Reactive oxygen species are important risk factors for age-related diseases, but they also act as signaling factors for endogenous antioxidative defense. The hypothesis that a multi-micronutrient supplement with nutritional doses of antioxidant nutrients and phytochemicals (MP) may provide protection against oxidative damage and maintain the endogenous antioxidant defense capacity was assessed in subjects with a habitually low intake of fruits and vegetables. In a randomized, placebo-controlled, and parallel designed trial, 89 eligible subjects were assigned to either placebo or MP for eight weeks. Eighty subjects have completed the protocol and included for the analysis. MP treatment was superior at increasing serum folate (*p* < 0.0001) and resistance to DNA damage (*p* = 0.006, tail intensity; *p* = 0.030, tail moment by comet assay), and LDL oxidation (*p* = 0.009) compared with the placebo. Moreover, the endogenous oxidative defense capacity was not weakened after MP supplementation, as determined by the levels of glutathione peroxidase (*p* = 0.442), catalase (*p* = 0.686), and superoxide dismutase (*p* = 0.804). The serum folate level was negatively correlated with DNA damage (*r* = −0.376, *p* = 0.001 for tail density; *r* = −0.329, *p* = 0.003 for tail moment), but no correlation was found with LDL oxidation (*r* = −0.123, *p* = 0.275). These results suggest that MP use in healthy subjects with habitually low dietary fruit and vegetable intake may be beneficial in providing resistance to oxidative damage to DNA and LDL without suppressing the endogenous defense mechanisms.

## 1. Introduction

No single theory can explain how aging and age-related diseases occur. Given that human cells are constantly exposed to an array of oxidizing agents present in the environment, one of the most popular explanations for aging and age-related pathologies is related to oxidative stress induced by the over-production of reactive oxygen species (ROS) [1,2]. Progress in understanding the deleterious effects of ROS on cells have shown that it is feasible to protect cells against oxidative damage through the free radical scavenging abilities of antioxidant micronutrient supplements [3]. However, some recent studies following the same paradigm have obtained opposing results [4,5]. The major distinction between these studies is generally the dose of antioxidant micronutrients administered [6], and these discrepancies may be explained by the fact that ROS not only cause damage to cells but also mediate diverse physiological responses that may contribute to normal cell function as well as to disease progression [7]. Thus, excessive doses of antioxidant micronutrients could abundantly scavenge ROS and interfere with beneficial ROS-dependent mechanisms. Therefore, it is necessary to establish the optimal doses of antioxidant micronutrients to ensure effective defense against ROS-induced damage while maintaining the minimum level of ROS needed to promote normal cell functions [8].

In parallel with these findings, epidemiological studies have revealed that the consumption of fruits and vegetables is associated with protective effects against age-related pathologies [9]. The possibility that the complex mixture of phytochemicals in fruits and vegetables may contribute to their preventive effects through activation of endogenous antioxidant defense systems has also been raised [10]. With this in mind, we hypothesized that a combination of nutritional doses of micronutrients and phytochemicals may constitute an effective tool to protect against the oxidative stress encountered in daily life while maintaining endogenous ROS homeostasis. To investigate this hypothesis, we conducted a double-blind, randomized, and placebo-controlled clinical trial to monitor the impacts of a multi-micronutrient supplement with nutritional doses of antioxidant nutrients and phytochemicals on the protection against oxidative damage to DNA and lipids in adults with a habitually low intake of fruits and vegetables. Furthermore, the impact of this dietary supplement on the maintenance of the endogenous antioxidant enzyme activities was also analyzed.

## 2. Experimental Section

### 2.1. Test Products

A multi-micronutrient supplement containing nutritional doses of antioxidant nutrients and phytochemicals (MP: Double X™) and a color-matched placebo (dextrose) were provided by Access Business Group LLC (Ada, MI, USA). This MP supplement (12 tablets per day) provides the following micronutrients: 14 vitamins (693 μg RE A, 200 IU D, 27.8 IU E, 55 μg K, 2 mg B_1_, 2.4 mg B_2_, 3 mg B_6_, 2 μg B_12_, 200 mg C, 26 mg niacin, 60 μg biotin, 3 mg β-carotene, 400 μg folate, and 10 mg pantothenic acid), and nine minerals (700 mg calcium, 10 mg iron, 75 μg iodine, 12 mg zinc, 1.5 mg copper, 2 mg manganese, 220 mg magnesium, 50 μg chromium, and 50 μg selenium). The supplement also contains phytochemicals from extracts or powders of acerola, alfalfa, brassica, carrot, citrus, grape seed, grape skin, rosemary, and spinach. High performance liquid chromatography (HPLC) analysis of the chemical signature revealed that the MP contained keracyanin, ellagic acid, hesperidin, rosmarinic acid, quercetin, and genistein, in abundance. Analysis of the antioxidant potency of the MP revealed 35% inhibition by 2,2-diphenyl-1-picrylhydrazyl (DPPH) [11], 2.8% inhibition by 2,2′-azino-di-(3-ethylbenzthiazoline-6-sulphonic acid) (ABTS) [12], 1,122 μmol Trolox/g in the oxygen radical absorption capacity (ORAC) assay [13], 2.0 mmol Fe^2+^/g in the ferric reducing antioxidant capacity (FRAP) assay [14], and 4.5 mg/g total phenol content as gallic acid equivalent by Singleton and Rossi’s modification of Folin-Ciocalteu’s colorimetric method [15].

### 2.2. Subjects

One hundred and five healthy adults (25–69 years old) were recruited from the Samsung Medical Center (Seoul, Korea). Eligible participants were adults with habitually low fruit and vegetable intake as screened by a diet quality score ≤36 (scale, 0–46). The diet quality score is based on reported consumption of foods bearing high amounts of antioxidant nutrients. A diet quality score ≤36 was chosen, as this is predictive of participants consuming less than the estimated adequate recommendations for antioxidant nutrients [16]. Exclusion criteria consisted of the regular use of multi-vitamin/multi-mineral supplements or medications (Coumadin, aspirin, hypertension medications, or other medications that influence hemostasis), hypertension (>160 mmHg systolic blood pressure or >100 mmHg diastolic blood pressure), alcohol intake (>64 g alcohol per day), known hypersensitivity to the study product, and pregnancy or lactation.

**Figure 1:**
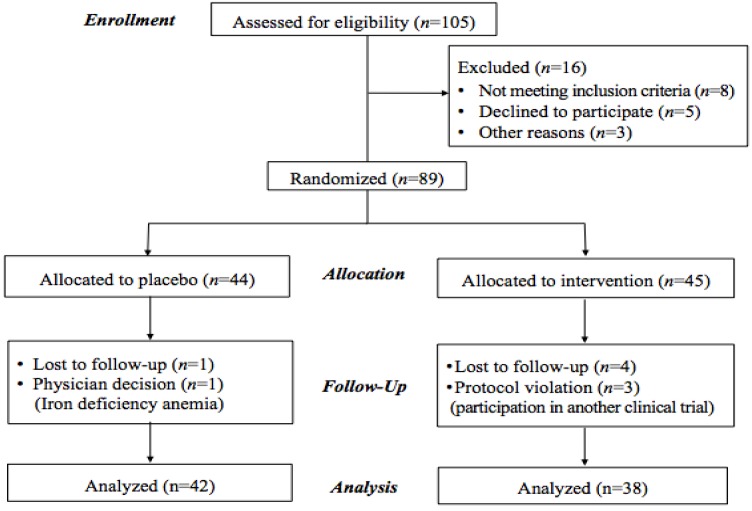
Flow diagram of the progress through the phases of a randomized trial.

Eighty-nine subjects were enrolled in the trial and received a baseline assessment and a complete blood count analysis. All study participants provided written informed consent before enrollment. The study protocols were approved by the Institutional Review Boards of Samsung Medical Center, and Ewha Womans University (Seoul, Korea). The study was also registered in the International Clinical Trials Registry Platform of the WHO with the following identification: KCT0000283. The results are reported according to Consolidated Standards of Reporting Trials guidelines [17] and a CONSORT flow chart (Figure 1) was included.

### 2.3. Study Design

This was a double-blind, randomized, parallel-arm, placebo-controlled, eight-week study. After a two-week lead-in period, the eligible participants were assigned to either the MP group (*n* = 45) or the placebo group (*n* = 44) using a stratified randomization to guarantee comparability of age and sex distribution between the two groups. The randomization code was prepared by a staff member who was not involved in running the trial, by using computer-generated random numbers. The group allocation was blinded for both investigators and participants. The principal investigator was provided with a sealed envelope for each subject, but asked to open after entering all data into the computer or in a medical emergency. Allocation concealment was maintained successfully as no sealed envelope was opened voluntarily or accidentally during the study.

At initiation of the study, the subjects received one bottle of the MP or placebo, which were made indistinguishable by their identical packaging. The subjects were asked to take six tablets, twice a day, consisting of a total of 12 tablets (5.1 g) a day for eight weeks, preferably with water after meals. The dose for the MP was chosen based on the commercially recommended level. The study product was dispensed three bottles at a time, which was enough for 17 days, thus, allowing for subject visits every 14 ± 3 days. At each visit, the remaining pills remained were counted by research coordinators. The subjects were excluded from the analysis if they consumed <80% of the recommended dose. The subjects were asked to report any possible adverse events.

During the study period, the subjects were asked to maintain their regular diet and lifestyle but to avoid certain foods that ranked high in antioxidant content. To assess nutrient intake and monitor dietary compliance, food quality scores and three-day food records (two weekdays and one weekend day) were collected both at the beginning and end of the study. Dietary energy values and nutrient contents were calculated using the Ccomputer Aided Nutritional analysis program (CAN-pro 3.0, Korean Nutrition Society, Seoul, Korea).

### 2.4. Outcome Measurements

At the beginning of the study and the end of the intervention, fasting venous blood was collected into the following vacutainers (Becton Dickinson, Franklin Lakes, NJ, USA): a clot activator tube (red-top) for serum analysis and a K_2_-ethylenediaminetetraacetic acid (EDTA) tube (lavender-top) for plasma, red blood cell (RBC), and peripheral blood mononuclear cell (PBMC) analysis. The samples were then centrifuged and transferred into plastic vials for further analysis. PBMCs were isolated from the buffy coat of EDTA-treated whole blood and washed three times with ice-cold phosphate buffered saline (PBS). RBCs were washed three times with ice-cold double-distilled water. Plasma, PBMCs, and RBCs were stored at −80 °C prior to analysis.

A single-cell gel electrophoresis (SCGE) assay was performed as described by Olive & Banath [18]. Briefly, PBMCs were spread on agarose-coated slides, lysed in a lysis solution (2.5 M NaCl, 0.01 M Tris, 0.1 M Na_2_EDTA, 1% (v/v) Triton X-100, and 10% (v/v) DMSO), electrophoresed at 31 V and 300 mA for 30 min, and then stained with 20 μg/mL ethidium bromide. The slides were evaluated under a fluorescence microscope (Nikon, Tokyo, Japan). Images from at least 50 comets on each slide were analyzed with COMET 4.2 image analysis software (Perceptive Instruments, Suffolk, UK). The amount of DNA damage was expressed as the tail intensity, tail length, and tail moment. Lymphocyte 8-hydroxy-deoxyguanosine (8-OH-dG) content was determined using a QIAamp DNA mini kit (Qiagen, Hilden, Germany) for DNA purification and an enzyme immunoassay (EIA) kit (Cayman, Ann Arbor, MI, USA) for quantitative detection. Plasma malondialdehyde (MDA) was quantified using a colorimetric assay kit (Oxford Biomedical Research, Oxford, MI, USA). Plasma oxidized low-density lipoprotein (ox-LDL) was analyzed using an enzyme-linked immunosorbent assay (ELISA) kit (Mercodia, Uppsala, Sweden). Serum folate was quantified with a direct chemiluminescent two-step sandwich immunoassay using a Bayer ADVIA Centaur Analyzer (Siemens Medical Solutions, Bohemia, NY, USA). Plasma transcobalamine I and II levels were determined by ELISA (Uscn Life Science Inc., Wuhan, China). The plasma β-carotene concentration was determined by HPLC methods with UV detection at 450 nm using a Capcell Pak C18 (MG, 5 μm, 4.6 × 250 mm, Shiseido). Glutathione peroxidase (GPx), superoxide dismutase (SOD), and catalase (CAT) activities were measured in RBCs using colorimetric assay kits (Cayman, Ann Arbor, MI, USA).

### 2.5. Sample Size Estimation and Statistical Analysis

**Figure 2:**
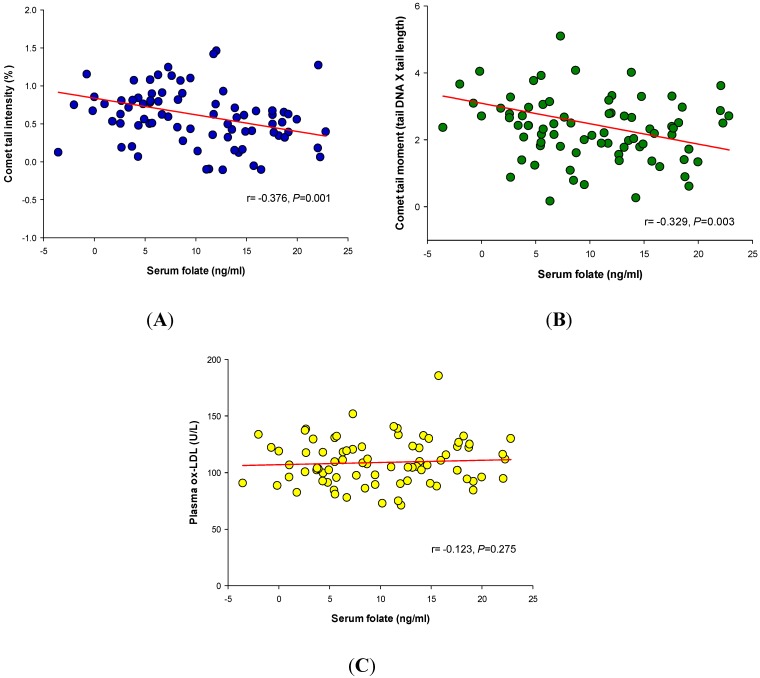
Relationship between the serum folate level and oxidative stress markers in all individuals at the end of the study, including comet tail intensity (**A**), comet tail moment (**B**), and plasma ox-LDL (**C**). The Pearson correlation coefficient (*r*-value) and *p*-value are shown adjacent to the line.

Assuming a 20% drop-out rate among the cases, the sample size was estimated at 45 subjects per group to provide a power of 80% to detect a difference in DNA tail intensity as the primary outcome of interest between the two groups, which is in agreement with an eight-week, randomized, double-blind, placebo-controlled, parallel study [19], with a two-sided α-level of 0.05. All results were analyzed according to the per-protocol principle. The interquartile range (IQR) was used to detect the presence of potential outliers. The observations that were outside of Q1 − (3 × IQR) and Q3 + (3 × IQR) were removed as outliers as follows: folate (1) and DNA tail intensity (1). A Kolmogorov-Smirnov test was used to assess the normality of each variable. Square root transformation was performed on skewed variables. The Student’s *t*-test or chi-square test was used to compare the baseline variables between groups. Repeated-measures analysis of variance (ANOVA) was undertaken to compare the nutritional composition of subjects’ diets between groups over time. To clarify the effect of MP supplementation by adjusting baseline status, stepwise multiple linear regression models were performed to investigate the association of each response variable with the baseline other variables (general characteristics, dietary intakes, oxidative stress biomarkers) and potential confounding variables were selected. The covariates that were deemed significant (*p* < 0.05) were retained. One-way analysis of covariance (ANCOVA) was used to compare the efficacy variables at baseline and at endpoint between the groups using the adjusted least squares (LS) mean values. Correlation was calculated with Pearson correlation coefficients. The data are presented in Table 1 and Table 2 as the means ± standard error (SE) and in Table 3 and Figure 2 as the adjusted LS means ± SE. Statistical analyses were performed using the Statistical Analysis Systems package version 9.3 (SAS Institute, Cary, NC, USA). A two-tailed value of *p* < 0.05 was considered statistically significant.

**Table 1:** Baseline characteristics of the subjects who completed the intervention trial ^1^.

Characteristics	Placebo (*n* = 42)	MP (*n* = 38)	*P*-Value ^2^
Age (year)	44 ± 2 ^3^	43 ± 2	0.918
Female/Male (*n*)	23/19	19/19	0.670
Body mass index (kg/m^2^)	24 ± 0.4	24 ± 0.5	0.918
Systolic blood pressure (mmHg)	122 ± 3	119 ± 2	0.474
Diastolic blood pressure (mmHg)	82 ± 3	78 ± 2	0.329
Serum triglycerides (mg/dL)	108 ± 8	141 ± 15	0.060
Serum total cholesterol (mg/dL)	180 ± 5	187 ± 4	0.226
Serum HDL-cholesterol (mg/dL)	55 ± 2	54 ± 3	0.534
Serum LDL-cholesterol (mg/dL)	102 ± 5	104 ± 4	0.694
Smoker/Non-smoker ^3^	8/34	7/31	0.076

^1^ Data are the means ± SE (all such values). MP, multi-micronutrient supplement with nutritional doses of antioxidant nutrients and plant ingredients; ^2^ The Student’s *t*-test or chi-square test (gender and smoking) was used to compare differences between groups; ^3^ Smokers were defined as subjects who used to smoke more than three cigarettes per day.

**Table 2:** Nutritional composition of the subjects’ diets throughout the study period ^1^.

Nutrients	Placebo (*n* = 42)		MP (*n* = 38)		*P*-Value ^2^
	Week 0	Week 8	Week 0	Week 8	
Diet quality score ^3^	23 ± 1.2	20 ± 1.1	19 ± 1.1	18 ± 1.0	0.448
Total energy (kcal) ^4^	1778 ± 69	1702 ± 56	1789 ± 68	1772 ± 65	0.587
Carbohydrate (g) ^4^	250 ± 10	237 ± 8	257 ± 10	252 ± 10	0.940
Fat (g) ^4^	49 ± 3.1	49 ± 2.9	50 ± 3.2	50 ± 3.0	0.557
Protein (g) ^4^	72 ± 3.0	71 ± 3.0	72 ± 4	74 ± 4	0.883
Fiber (g)	18 ± 0.8	17 ± 0.9	17 ± 1.0	16 ± 0.9	0.511
β-Carotene (mg) ^4^	3.2 ± 0.2	2.6 ± 0.2	1.9 ± 0.2	4.8 ± 0.2	<0.0001
Vitamin C (mg) ^4^	72 ± 5.1	64 ± 4.1	63 ± 5.5	243 ± 4.4	<0.0001
Vitamin E (mg) ^4^	14 ± 1.0	14 ± 0.8	13 ± 0.9	58 ± 1.1	<0.0001
Folate (μg) ^4^	227 ± 13	211 ± 11	213 ± 18	570 ± 13	<0.0001
Zinc (mg) ^4^	8.2 ± 0.4	8.3 ± 0.4	8.2 ± 0.5	20 ± 0.5	<0.0001
Iron (mg) ^4^	14 ± 0.7	15 ± 2.7	13 ± 0.8	22 ± 0.9	<0.0001

^1^ All values are the means ± SE. Intake levels were estimated from three-day food records using CAN-pro (Korean Nutrition Society, Seoul, Korea). The intake of the test product was included in the analysis. MP, multi-micronutrient supplement with nutritional doses of antioxidant nutrients and plant ingredients; ^2^ Repeated-measures ANOVA was used to test the difference for group (Placebo and MP) × week (Week 0 and Week 8); ^3^ Diet quality score was measured using a validated scoring method [16]; ^4^ Data were square-root transformed for normalization.

**Table 3:** Summary of oxidative stress biomarkers at baseline and after the eight-week intervention ^1^.

Biomarkers	Placebo (*n* = 42)		MP (*n* = 38)		*P-Value* ^2^	
	Week 0	Week 8	Week 0	Week 8	Week 0	Week 8
Exposure biomarkers to MP						
β-Carotene (μg/mL)	203 ± 18	188 ± 16	219 ± 19	240 ± 17	0.606	0.081
Folate (ng/mL) ^3^	8.7 ± 0.5	8.6 ± 0.7	8.2 ± 0.6	20 ± 0.7	0.863	<0.0001
Transcobalamin I (pg/mL)	1157 ± 469	1526 ± 100	1159 ± 494	1646 ± 111	0.841	0.284
Transcobalamin II (pg/mL)	1.0 ± 0.2	1.3 ± 0.2	1.1 ± 0.2	1.4 ± 0.2	0.216	0.880
Peroxidation products						
DNA damage						
Tail intensity (%) ^3^	0.5 ± 0.0	0.7 ± 0.1	0.4 ± 0.1	0.3 ± 0.1	0.379	0.006
Tail length (μm)	7.4 ± 0.3	6.3 ± 0.2	7.1 ± 0.3	6.5 ±0.2	0.370	0.306
Tail moment	1.3 ± 0.2	1.8 ± 0.2	1.2 ± 0.2	0.9 ± 0.2	0.479	0.030
8-Hydroxy-2′-deoxyguanosine (pg/μg DNA)	4.4 ± 0.4	4.1 ± 0.3	5.1 ± 0.4	4.1 ± 0.3	0.471	0.951
Malondialdehyde (nmol/mL)	2.3 ± 0.1	1.8 ± 0.1	2.4 ± 0.1	1.8 ± 0.1	0.588	0.790
Oxidized LDL (U/L)	48 ± 1.6	64 ± 4.0	50 ± 1.8	53 ± 4.4	0.636	0.009
Endogenous oxidative defense capacities						
Glutathione peroxidase (nmol/min/mL)	83 ± 3.1	72 ± 2.9	85 ± 3.3	58 ± 3.1	0.170	0.442
Catalase (nmol/min/mL)	23 ± 0.2	23 ± 0.2	23 ± 0.2	23 ± 0.2	0.953	0.686
Superoxide dismutase (U/L)	11 ± 0.3	10 ± 0.2	11 ± 0.3	10 ± 0.2	0.480	0.804

^1^ All values are the adjusted LS means ± SE. Data were square-root transformation for normalization. MP, multi-micronutrient supplement with nutritional doses of antioxidant nutrients and plant ingredients; ^2^ One-way ANCOVA was used to test the differences between groups with a subset of covariates selected using stepwise multiple linear regression; ^3^ Values after excluding outlier data: DNA tail intensity (1) and folate (1).

## 3. Results

Of 89 eligible subjects, 80 (38 in the MP group and 42 in the placebo group) completed the eight-week supplementation for inclusion in the analysis (Figure 1). Four subjects withdrew during the intervention due to iron deficiency anemia (one in the placebo group) or participation in another clinical trial (three in the MP group). Five subjects (four in the MP group and one in the placebo group) were lost to follow-up. The mean compliance ratio for all subjects was 92.4%, with no subject excluded due to insufficient compliance. Mild to moderate adverse symptoms (*i.e.*, headache, abdominal discomfort, constipation, sore throat, and stomach flatulence) were reported in both groups. The differences in the incidence of adverse symptoms between the two groups were not significant. There were no significant subjective symptoms or serious adverse events reported by any of the participants in this study.

All subjects were documented to fit the protocol, and there was no significant difference between the two treatment groups according to baseline characteristics (Table 1). All subjects represented adults with a low habitual intake of fruits and vegetables from baseline to the completion of the study, as reflected by a mean diet quality score ≤36 (Table 2). The intake levels of total energy and selected nutrients were not different between the groups at baseline, with the exception of β-carotene consumption. While the total energy and macronutrient intake levels were unchanged during the experiment, micronutrient intake was significantly increased following MP supplementation (*p* < 0.0001).

The values for exposure, oxidative damage, and endogenous oxidative defense at baseline and at the end of the study, expressed as the adjusted LS mean ± SE, are summarized in Table 3. Overall, there was no significant difference between the groups at baseline. To assess exposure, we measured the levels of serum folate, plasma β-carotene, and transcobalamin I and II. As expected, compared with the placebo group, the MP group showed a clear increase in folate (1.1% decrease in placebo *versus* 143.3% increase in MP, *p* < 0.0001), a moderate increase in β-carotene (7.4% decrease in placebo *versus* 9.6% increase in MP, *p* = 0.081), and no change in the levels of transcobalamin I and II.

Next, to assess oxidative damage, we measured the peroxidation products of lipids and DNA by determining the levels of MDA, ox-LDL, 8-OH-dG, and three comet parameters. We observed significant resistance against oxidative damage in the MP group compared with the placebo group regarding DNA tail intensity (30% increase in placebo *versus* 26% decrease in MP, *p* = 0.006), DNA tail moment (39% increase in placebo *versus* 20% decrease in MP, *p* = 0.030), and ox-LDL (32% increase in placebo *versus* 7% increase in MP, *p* = 0.009). DNA tail length also tended to decrease more in the MP group, but this difference did not reach statistical significance. There was also no significant overall change in 8-OH-dG and MDA.

Last, we measured the activities of antioxidant enzymes in red blood cells to evaluate the impact of MP treatment on endogenous ROS homeostasis. None of the markers of endogenous antioxidant enzyme activities were significantly affected by MP supplementation, as determined by GPx (*p* = 0.442), CAT (*p* = 0.686), and SOD (*p* = 0.804).

Figure 2 shows the relationship between serum folate and DNA tail intensity, DNA tail moment, and plasma ox-LDL after the completion of the eight-week intervention. In all 80 individuals, irrespective of the treatment group, an expected inverse correlation was found between serum folate and DNA tail intensity (*r* = −0.376, *p* = 0.001) and between serum folate and DNA tail moment (*r* = −0.329, *p* = 0.003). There was, however, no correlation between serum folate and plasma ox-LDL (*r* = −0.123, *p* = 0.275).

## 4. Discussion

This study showed that a multi-micronutrient supplement that provides nutritional doses of antioxidant nutrients with phytochemicals might be effective in preventing oxidative damage while maintaining endogenous ROS homeostasis. Although all individuals are exposed to oxidative stress in daily life, different population groups differ in terms of their ROS level [8]. Therefore, identifying groups that are at risk of oxidative stress is important, and a considerable amount of data supports the notion that the level of fruit and vegetable intake is inversely correlated with oxidative stress in healthy subjects [16,20,21]. In this study, therefore, we selected a target group of adults who had a dietary habit of consuming low levels of fruits and vegetables using a scoring method. This diet quality score was previously validated for measuring overall diet quality to determine the associations between whole foods and oxidative stress in Korean adults by our research group [16]. It should also be emphasized that the dose of antioxidant nutrients in MP followed the recommended dietary allowances (RDAs) for Korean population. In the case of antioxidant nutrients, it is important to differentiate the pharmacological dose from the nutritional dose: the proper nutritional dose should follow the RDAs [22]. Subjects receiving the placebo showed a tendency for increased levels of DNA and LDL damage during the eight-week experimental period, whereas subjects receiving MP showed significant resistance to oxidative stress.

Many indices have been developed to measure oxidative stress. Among these, the most commonly used marker is the measurement of relatively stable peroxidation products [23]. For this study, we selected the comet assay and the quantitative measurement of 8-OH-dG in DNA for DNA damage as well as the fasting plasma levels of thiobarbituric acid-reacting substances (TBARS) and oxidized forms of LDL (ox-LDL) for lipid peroxidation. Regarding DNA damage, we observed significant improvement in the MP group with respect to the comet parameters. The comet assay is a technique for measuring and analyzing DNA breakage in mammalian cells. If the DNA strands contain breaks, the DNA supercoils become relaxed, and the broken ends are able to migrate during electrophoresis, thus allowing the quantification of DNA damage by measuring the displacement between the comet head containing the genetic material of the nucleus and the comet tail containing the leading ends of migrating fragments using computerized image analysis [18]. The most beneficial feature of the comet assay is its sensitivity for detecting low levels of DNA damage, which provide an indication of recent exposure at an early stage when the DNA could still undergo repair [24]. The tail length, % of DNA in the comet tail (tail intensity), and tail moment are the most frequently used comet parameters [25]. The tail intensity and tail moment are generally more reliable indicators of DNA damage than tail length because the extent to which the DNA loops extend from the comet head may be independent of increasing levels of DNA damage due to saturation as well as DNA supercoil structures [26,27]. In contrast, although the determination of 8-OH-dG obtained from the nuclear DNA of lymphocytes has been reported for comparative human monitoring studies [28], the differences were not high enough in this study to detect the effects of supplementation. Regarding lipid peroxidation, we failed to detect differences in plasma MDA concentrations between the two groups throughout the study period, whereas the level of ox-LDL in the plasma was shown to be sensitive enough to monitor changes that occurred during the eight-week study period. This lack of consistency may result from methodological problems inherent to the current approach for analyzing the amount of MDA in plasma. In particular, MDA measurement involves a reaction with TBA to produce a compound that can be detected by a UV-spectrophotometer, and then, TBA reacts with MDA, as well as with other non-lipid compounds, to generate nonspecific compounds that lie within the same absorption spectra [29]. The amount of MDA and 8-OH-dG can be estimated by the HPLC methods with a fluorescence detector [30] and an electrochemical detector [31], respectively. The HPLC method is generally more accurate than the spectrophotometric analysis that was used to quantify MDA and 8-OH-dG from blood samples in this study.

The other category of markers used to evaluate oxidative stress consists of an array of powerful cellular macromolecules that protect cells against peroxidation [23]. Antioxidant enzymes, such as SOD, CAT, and GPx, are included in this category as they act to decompose ROS. Interestingly, however, recent data have suggested that a low grade of oxidative stress is needed to maintain a certain level of endogenous antioxidants, which leads to increased resistance to subsequent higher levels of oxidative stress and damage. As a result, it was suggested that the administration of excessive doses of antioxidants might abolish ROS production and dangerously interfere with endogenous protective functions [8,32]. Many prospective clinical trials have found no health-promoting effects of antioxidants at high doses above RDA. Supplementation of vitamin C (500 mg/day), vitamin E (600 IU/day), and β-carotene (50 mg/day) showed no significant overall effects on the risk of type 2 diabetes in women at high risk of cardiovascular disease [33] and in the primary prevention of total cancer incidence or cancer mortality [34]. In the Selenium and vitamin E Cancer Prevention Trial (SELECT), Lippman *et al.* [35] reported that supplementation of selenium (200 μg/day) or vitamin E (400 IU/day), alone or in combination, did not reduce the risks of prostate cancer and other cancers. This line of thinking may explain the current controversies regarding the efficacy of multi-micronutrient supplements as antioxidants, as studies applying comparably high doses of antioxidants have reported negative results despite many other studies supporting the protective effects of micronutrients against oxidative stress [6,36]. In a more recent study, Ristow *et al*. [5] reported that dietary supplementation with a combination of high-dose vitamin C (1000 mg/day) and vitamin E (400 IU/day) significantly reduced exercise-induced oxidative stress in healthy subjects but also blocked the capacity for exercise-induced endogenous antioxidant defense. In contrast, in the present study, we found that daily MP consumption for eight weeks provided favorable changes against oxidative damage without suppressing endogenous antioxidant enzyme activities. It should be noted that the MP used in this study contains vitamins, minerals, and phytochemicals at approximately the current recommended dietary intake levels for Korean adults. Moreover, the dose and combination of multi-micronutrient supplements are important for balancing protection against oxidative damage and the maintenance of the endogenous defense system.

Direct estimation of nutrients and phytochemicals in the plasma is needed to identify the nutrients and phytochemicals responsible for a given health outcome. Consistent with the existing evidence [37,38], we found a statistically significant inverse relationship between the serum folate concentration and DNA stability by gel electrophoresis. In contrast, the serum folate concentration was not associated with the plasma ox-LDL levels. One interpretation of these results is that the protective effect of MP on LDL may be attributed to other components, including phytochemicals. Numerous investigations have suggested that a variety of phytochemicals found in plant-based foods and ingredients can help protect LDL [10]. For this study, however, it was difficult to determine the phytochemicals in the plasma due to chemical diversity [10]; more than 5000 individual phytochemicals have been identified in foods, and almost every phytochemical is further transformed into various metabolites in the systemic circulation after ingestion [39]. Indeed, this highlights the limitation of the current study. Cutting-edge technologies, such as high-throughput analytical approaches and bioinformatics, need to be applied to explore the relationships between phytochemical intake and metabolism and health.

## 5. Conclusions

The present study represents, to the best of our knowledge, the first attempt to confirm the hypothesis that a multi-micronutrient supplement with nutritional doses of antioxidant nutrients and phytochemicals may provide favorable protection against oxidative damage without suppressing the levels of endogenous antioxidant enzymes in healthy subjects. However, several limitations of this study must be noted. The observed ability of MP treatment to protect DNA stability is likely attributed to, at least in part, the significant increase in the serum folate concentration from 8 ng/mL to 20 ng/mL. However, the mechanisms of resistance to oxidative damage by MP supplementation are incompletely understood, as other vitamins, minerals, phytonutrients, and their metabolites in the blood were not measured in this study. Additionally, this study was designed to compare the intervention effects of the MP and the placebo, and this simple design could not provide a test to identify whether the benefits of MP supplementation could be attributed to the multi-micronutrients, the phytochemicals, or both. Within the limitations of this study, however, the results still offer empirical support for the use of a nutritional dose of multi-micronutrient supplementation with plant ingredients for adults habitually low in fruit and vegetable intake to improve DNA and LDL stability while maintaining ROS homeostasis. Nevertheless, further studies are needed to identify the compounds and mechanisms responsible for the observed effects.
